# Error in Table 2 Footnote

**DOI:** 10.1001/jamanetworkopen.2026.36401

**Published:** 2026-09-01

In the Original Investigation titled “Acute Respiratory Tract Infections and Severe Disease Among Hospitalized Children,”^1^ published June 9, 2026, the phrases *plasma exchange* and *respiratory support* were missing in footnote g of Table 2. This article has been corrected.^1^
